# Supplementary material for: Metasurface-stabilized optical microcavities
Source: Nat Commun. 2023 Feb 27;14:1114. doi: 10.1038/s41467-023-36873-7 (PMC9971257; doi:10.1038/s41467-023-36873-7)
Supplement: Supplementary file 1 — Supplementary Information [file 41467_2023_36873_MOESM1_ESM.pdf]

Supplementary Information for

Metasurface-Stabilized Optical Microcavities

Marcus Ossiander[1, *], Maryna Leonidivna Meretska[1], Sarah Rourke[1,2], Christina Spägele[1],

Xinghui Yin[1], Ileana-Cristina Benea-Chelmus[1,3], Federico Capasso[1, *]

1 John A. Paulson School of Engineering and Applied Sciences, Harvard University, 29 Oxford St, Cambridge, MA 02138, USA

2 University of Waterloo, Waterloo, ON N2L 3G1, Canada

3 Hybrid Photonics Laboratory, École Polytechnique Fédérale de Lausanne, Lausanne, CH-1015, Switzerland

* Corresponding Authors: mossiander@g.harvard.edu, capasso@seas.harvard.edu

Supplementary Figures

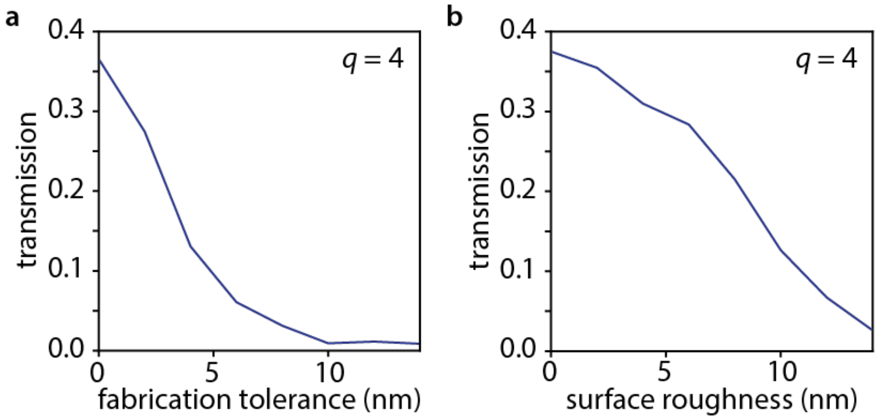

Supplementary Figure 1

Sensitivity of a Metasurface-Stabilized Cavity to Metasurface Pillar Diameter Fabrication Tolerance and Surface Roughness

a) modeled transmission at the resonance length of the mode with longitudinal mode index $q = 4$ for the cavity in Fig. 2a versus the standard deviation of the diameters of the metasurface nanopillars from the design diameters.

b) modeled transmission at the $q = 4$ resonance length for the cavity in Fig. 2a versus the surface roughness of the walls of the metasurface nanopillars.

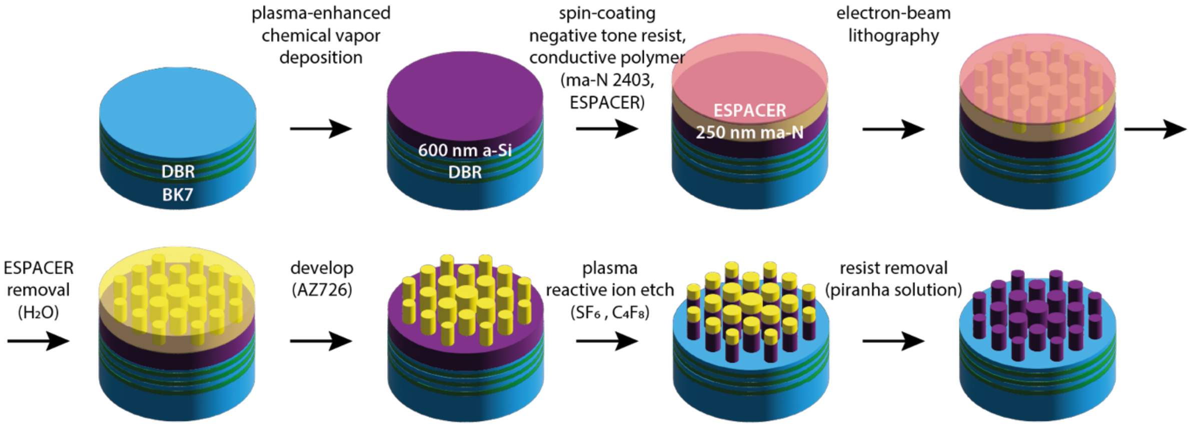

Supplementary Figure 2

Fabrication of metasurface microcavity stabilizers

We use a commercially available distributed Bragg reflector (DBR, light blue/green) on a 3 mm thick BK7 substrate (light blue) as our base material (Eksma 031-1550-i0). Using plasma-enhanced chemical vapor deposition, we deposit a 600 nm thick film of amorphous silicon (a-Si, purple) on the DBR. We then spin coat 250 nm of negative tone electron-beam lithography resist (Micro Resist Technology ma-N 2403, transparent yellow) and a thin layer of conductive polymer (Showa Denko ESPACER 300, pink) on the amorphous silicon layer. Via electron-beam lithography (Elionix HS-50), we write the metasurface design (solid yellow) into the resist before removing the conductive polymer using water and remove unexposed resist using developer (MicroChemicals AZ726 MIF). Amorphous silicon is then removed in areas that are no longer covered by resist using an inductively coupled plasma reactive ion etch (SF_6, C_4F_8) before all remaining resist is removed using Piranha solution.

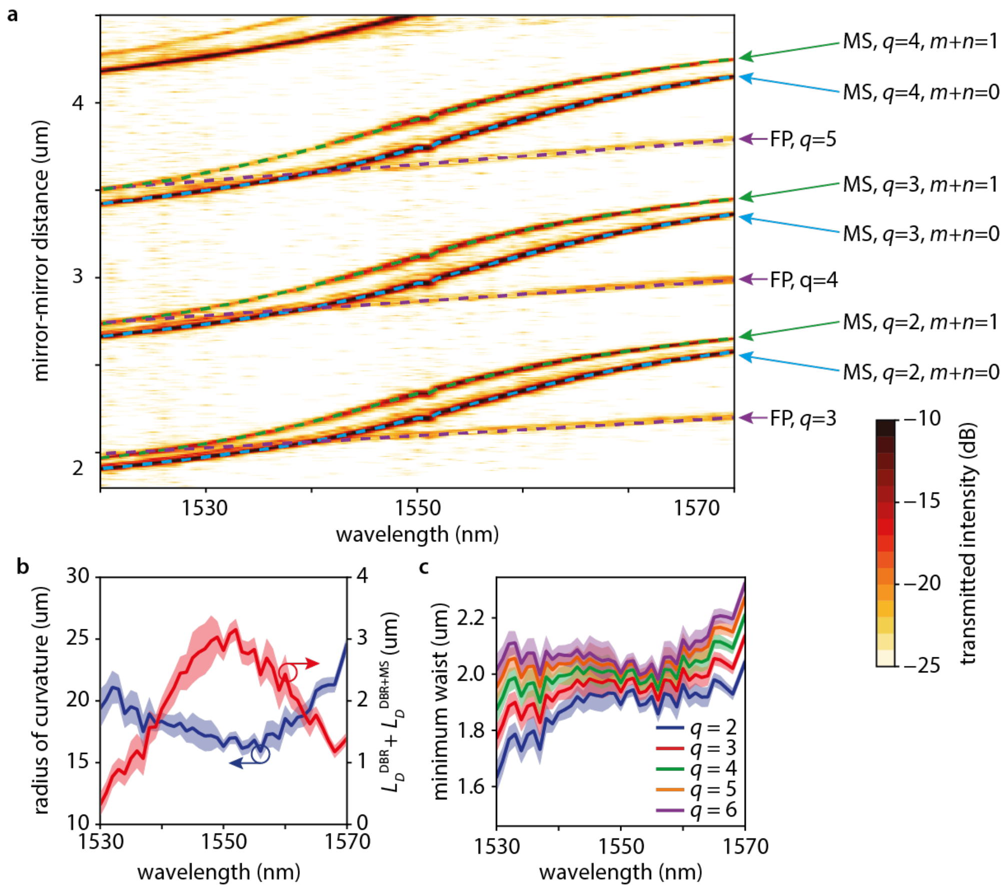

Supplementary Figure 3

Transverse confinement characterization in the metasurface-stabilized optical microcavity.

a) measured wavelength- and cavity length-dependent transmission (normalized) of the metasurface-stabilized microcavity (MS, false color plot). To examine modes with transverse mode index $n + m = 1$ (marked with dashed green lines) we shift the minimum beam waist of the incoming light beam along the propagation direction. This increases the mode overlap of the incoming beam with these higher modes compared to the fundamental transverse modes with $n + m = 0$ (marked with dashed blue lines). Unstable modes in the Fabry-Perot cavity (FP) around the metasurface area are faintly visible (marked with dashed purple lines). q denotes the longitudinal mode index.

b) wavelength-dependent effective radius of curvature of the metasurface (blue line) and wavelength-dependent modal penetration depth sum $L_D^{\mathrm{DBR}} + L_D^{\mathrm{DBR+MS}}$ (red line) of a distributed Bragg reflector (DBR) and the metasurface on a DBR (DBR+MS). Shaded areas denote standard deviations calculated from the fits of the positions of the resonances with $n + m = 0$ and $n + m = 1$.

c) wavelength and longitudinal mode index-dependent minimum waist (located at the cavity end mirror without metasurface) of the metasurface-stabilized cavity modes. Shaded areas denote standard deviations calculated via propagation of the standard deviations in panel b.

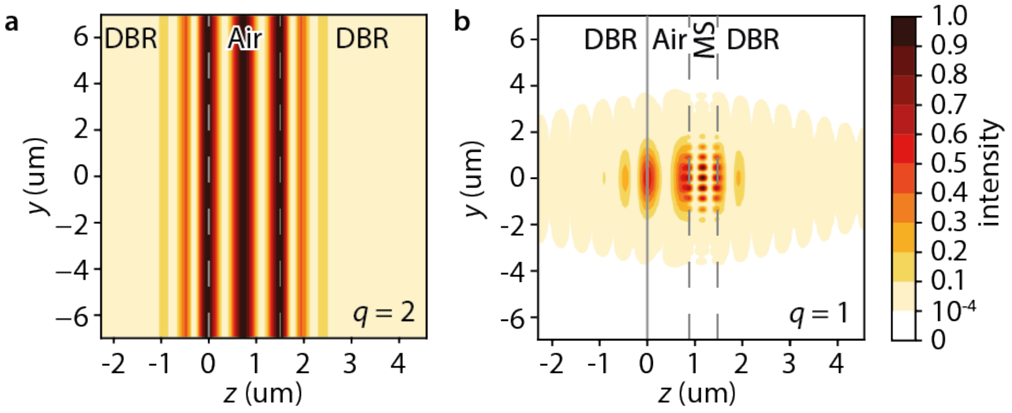

Supplementary Figure 4

Mode Counting and Comparison of a Planar-Planar and a Metasurface-Stabilized Cavity

a) light intensity distribution of the mode with longitudinal mode index $q = 2$ in a Fabry-Perot cavity built from two planar low-index terminated distributed Bragg reflectors (DBRs). y is a transverse spatial coordinate, z is the longitudinal spatial coordinate. Color bar in panel b.

b) light intensity distribution of the mode with longitudinal mode index $q = 1$ in a metasurface (MS) stabilized microcavity using the manufactured design.

Supplementary Methods

Supplementary Method 1

Transverse Confinement

To determine the transverse confinement of light in the metasurface-stabilized cavity, we move the minimum waist position of the incident light beam along the propagation direction. This increases the mode overlap of the transverse modes with $n + m = 1$ compared to those with $n + m = 0$. The Gouy phase $\theta_{nm}^{\text{Gouy}}$ of a Hermite-Gaussian beam is a phase shift that a focused mode experiences relative to a plane wave when it propagates through its focus (see main text). Because the inverse tangent function is limited to $(-\pi/2, \pi/2)$, the overall magnitude of the Gouy phase acquired by a mode is determined by its transverse mode numbers $m + n$ (see Equation (3)). However, most of the phase accumulates within the Rayleigh range around a mode's minimum beam waist (focus). Therefore, because stronger focusing decreases the Rayleigh range, it also causes a faster accumulation of the Gouy phase around the minimum beam waist. Because the transverse mode numbers influence the magnitude of the Gouy phase a mode acquires (see Equation (3)), it changes a mode's resonance length. The resultant resonance length difference $\Delta L_q(\lambda) = L_{q,n+m=1}(\lambda) - L_{q,n+m=0}(\lambda)$ between the resonance lengths of the fundamental transverse modes $L_{q,n+m=0}$ and the first excited transverse modes $L_{q,n+m=1}$ allows us to extract the wavelength-dependent effective radius of curvature of our metasurface. For this purpose, we interpret the metasurface as a curved mirror with an effective radius of curvature R_{MS}. For a given metasurface and wavelength, R_{MS} is constant and should match the radius of curvature of any resonant cavity mode. Therefore, we require $R_{\text{MS}} = R(L_{\text{cav}}) = L_{\text{cav}}\left(1 + \frac{z_R^2}{{L_{\text{cav}}}^2}\right)$ to derive

$$\frac{L_{\text{cav}}}{z_R} = \frac{1}{\sqrt{\frac{R_{\text{MS}}}{L_{\text{cav}}} - 1}}. \tag{16}$$

We then use the resonance conditions and the resonance lengths for the first excited transverse modes $L_{\text{cav}} = L_{q,n+m=1}$ and the fundamental transverse modes $L_{\text{cav}} = L_{q,n+m=0}$ and allow different Rayleigh ranges $z_{R,q,n,m}$ for the modes:

$$2\pi q = 2\theta_{n,m=0}\big(0,0,L_{q,n+m=0}\big) = 2\theta_{n+m=1}\big(0,0,L_{q,n+m=1}\big) = \\ -kL_{q,n+m=0} + \tan^{-1}\left(\frac{L_{q,n+m=0}}{z_{R,q,n+m=0}}\right) = -kL_{q,n+m=1} + 2\tan^{-1}\left(\frac{L_{q,n+m=1}}{z_{R,q,n+m=0}}\right). \tag{17}$$

Combining (17) with $\Delta L_q = L_{q,n+m=1} - L_{q,n+m=0}$ leads to $\Delta L_q = \frac{\lambda}{2\pi}\left(2\tan^{-1}\left(\frac{L_{q,n+m=1}}{z_{R,q,n+m=0}}\right) - \tan^{-1}\left(\frac{L_{q,n+m=0}}{z_{R,q,n+m=0}}\right)\right)$. We then replace $\frac{L_{q,n+m=0}}{z_{R,q,n+m=0}}$ and $\frac{L_{q,n+m=1}}{z_{R,q,n+m=0}}$ and introduce R_{MS} using Equation (16). The penetration of the mode into the cavity mirrors adds an additional Gouy phase to the phase accumulated by the mode in between the cavity mirrors. Therefore, we introduce the modal penetration depths for a planar DBR L_D^{DBR} and the metasurface on the DBR $L_D^{\mathrm{DBR+MS}}$, and replace $L_{q,n,m} \to L_{q,n,m} + L_D^{\mathrm{DBR}} + L_D^{\mathrm{DBR+MS}}$ in the Gouy phase to obtain the result:

$$\Delta L_q = L_{q,n+m=1} - L_{q,n+m=0} = \frac{\lambda}{2\pi}\left(2\tan^{-1}\left(\frac{1}{\sqrt{\frac{R_{\mathrm{MS}}}{L_{q,n+m=1}+L_D^{\mathrm{DBR}}+L_D^{\mathrm{DBR+MS}}}-1}}\right) - \tan^{-1}\left(\frac{1}{\sqrt{\frac{R_{\mathrm{MS}}}{L_{q,n+m=0}+L_D^{\mathrm{DBR}}+L_D^{\mathrm{DBR+MS}}}-1}}\right)\right). \quad (18)$$

Wavelength and cavity length-dependent transmission data is presented in Supplementary Fig. 3a. We observe wavelength-dependent resonance length differences $\Delta L_q(\lambda)$ which are explainable by a wavelength-dependent effective radius of curvature and a wavelength-dependent modal penetration depth. Fitting $\Delta L_q(\lambda)$ allows us to extract both the effective radius of curvature of the metasurface and the sum of the modal penetration depths into the planar and metasurface covered DBR $L_D^{\mathrm{DBR}} + L_D^{\mathrm{DBR+MS}}$, which are plotted in Supplementary Fig. 3b. Because the effective radius of curvature of the metasurface corresponds to that of the cavity mode's wavefront, this measurement also gives the minimum waist diameters of the cavity modes, shown Supplementary Fig. 3c.
